# Predictors for earlier return to work of cancer patients

**DOI:** 10.1007/s11764-017-0655-7

**Published:** 2017-10-26

**Authors:** M. D. J. Wolvers, M. C. J. Leensen, I. F. Groeneveld, M. H. W. Frings-Dresen, A. G. E. M. De Boer

**Affiliations:** Academic Medical Center, Department: Coronel Institute of Occupational Health, Amsterdam Public Health Research Institute, Room K0-115, Meibergdreef 15, 1105 AZ Amsterdam, The Netherlands

**Keywords:** Return to work, Fatigue, Chemotherapy, Work ability, Self-efficacy, Occupational health

## Abstract

**Purpose:**

This study aims to investigate how perceived work ability, job self-efficacy, value of work, and fatigue predict return to work (RTW) in cancer patients who received chemotherapy.

**Methods:**

Data of a before-after study on a multidisciplinary intervention that aimed to enhance RTW was used, consisting of four assessments up to 18 months. Time to partial and full RTW of 76 and 81 participants, respectively, was analyzed in Cox proportional hazard analysis with time-dependent variables. Univariate analyses of work ability, job self-efficacy, value of work, or fatigue as covariates were succeeded by multivariate analyses of work ability and either job self-efficacy, value of work, or fatigue as covariates.

**Results:**

Participants were mostly female (93%), and diagnosed with breast cancer (87%). Most participants were permanently employed (84%) and 48% was sole breadwinner. When adjusted for timing variables and prognostic factors, all hypothesized factors were predictive for earlier RTW (*p* < .05). In models that also included work ability, only job self-efficacy significantly predicted earlier full RTW: hazard ratio = 1.681; *p* = .025.

**Conclusions:**

Lower fatigue and higher value of work, work ability, and job self-efficacy of cancer survivors are associated with earlier RTW. Work ability and job self-efficacy seem to be key predictors.

**Implications for cancer survivors:**

Limiting fatigue, increasing value of work, job self-efficacy, and perceived work ability are promising goals for enhancing earlier RTW. Occupational rehabilitation should empower patients to organize appropriate conditions for work and to educate them on rights and obligations during sick leave.

**Electronic supplementary material:**

The online version of this article (10.1007/s11764-017-0655-7) contains supplementary material, which is available to authorized users.

## Introduction

Many cancer patients strive to return to work (RTW) when cancer treatment has ended. Returning to work can provide cancer patients with a sense of structure, helps in establishing identity, and contributes to partaking in social connections [1]. Also, working is associated with feelings of returning to normality [2].

However, smooth work resumption is not self-evident for cancer survivors. In a review study in a mixed sample of cancer survivors, 62% had returned to work after 12 months, and the average sick leave was 151 days [3]. Correspondingly, unemployment risk of cancer survivors in general is 1.4 times that of healthy controls [4].

Strong evidence exists that physical exertion, less invasive surgery, chemotherapy, and cancer site are prognostic factors for RTW [5]. However, those factors are largely fixed, whereas knowledge on factors that are potentially modifiable could help to direct interventions aimed at enhancing RTW. Therefore, this study focuses on variables that can be intervened on during or after cancer treatment. Several factors have shown to be relevant in this perspective, among which perceived work ability, job self-efficacy, value of work, and fatigue.

Perceived work ability is a central concept in evaluating the perspective of patients in an occupational context. It is a predictor of time to RTW [6] and for work continuation [7] in cancer survivors, but is largely refrained from situational or external factors that either push or prohibit actual RTW. Also, it reflects the work ability in the context of a patient’s job without framing which aspects are important for their specific job. This contrasts measures of job self-efficacy. Job self-efficacy [8] is a more comprehensive way of assessing a person’s perception of their work-related capabilities, structured by presenting items on specific tasks and situations that a person could encounter when they would be at work. Job self-efficacy of patients is cross-sectionally related with lower levels of sick leave in colorectal cancer patients, but not predictive for sick leave 6 months later [9].

A cancer diagnosis can lead to a re-evaluation of the importance of work [10, 11]. A positive attitude toward work may benefit work resumption, as it was a supportive factor for work performance in a qualitative study among patients with different types of chronic disorders [12]. However, value of work or changes thereof have to the best of our knowledge not yet been studied in cancer survivors specifically, nor for predicting time to RTW.

Fatigue is a common and debilitating side effect of cancer and cancer treatment [13]. It is considered a multidimensional symptom (“physical, emotional and/or cognitive tiredness or exhaustion” are mentioned in a commonly-used definition (Berger et al. [14])), but also unidimensional measures are frequently used and valid [15]. Cognitive fatigue [16] and more general measures of fatigue [17] affect work ability. Fatigue is named as an important problem at work [17] and was called “the main factor impeding RTW” in six qualitative studies [18]. Fortunately, effective treatments exist [19], including physical exercise training, which has shown to limit or decrease fatigue symptoms [20, 21] and is acceptable to perform during chemotherapy [22]. Counterintuitively, the relation of fatigue and time to RTW is not evident: lower fatigue was related with earlier work resumption in univariate models [23, 24], but not in multivariate models that also included work ability or treatment modalities [6, 24, 25].

In this paper, the associations of work ability, job self-efficacy, value of work, and fatigue with time to RTW will be studied to improve understanding of and rationale for interventions directed at enhancing work resumption. Up to now, these variables were mostly studied as if they were fixed factors: measurements at baseline or 6 months after diagnosis were used to predict RTW up to 5 years post diagnosis. This study will explicitly acknowledge the fluctuating character of these four variables by using three assessments in the course of 1 year of participants who participated in an intervention aimed at targeting all four of the factors to enhance RTW.

As work ability is expected to be a central concept for returning to work, we will also study how work ability relates to job self-efficacy, value of work, and fatigue in the context of RTW. Therefore, we are especially interested if job self-efficacy, value of work, and fatigue have additional predictive value for RTW over work ability, or that work ability “covers” their shared variance already.

### Objective

The objective of this study was to assess which of the factors that were targeted in a multidisciplinary intervention to facilitate RTW are related to time to partial and full RTW. Firstly, we will investigate whether higher perceived work ability, job self-efficacy, value of work, and lower fatigue predict earlier RTW. Secondly, we will study whether job self-efficacy, value of work, and fatigue have additional predictive value for RTW compared to, or over, perceived work ability.

## Methods

For this study, data of a before-after study on a multidisciplinary intervention that aimed to enhance RTW [26] was used to predict time to RTW by means of survival analyses [27].

### Participants

Participants were recruited in two large medical centers in the Netherlands. Patients were eligible when they were aged between 18 and 60 years, had a primary diagnosis of cancer, and were being or would soon be treated with chemotherapy with curative intent. Eligible patients had been in paid employment at the time of diagnosis and were absent from work or intended to report sick before the start of treatment. Exclusion criteria were having testicular cancer, or severe mental disability, or being physically unable to perform exercise training [26]. An additional criterion for the current study was that participants had actually reported sick or were partially sick-listed at the time of the baseline assessment. Ethical approval was granted from the AMC medical ethics committees. Informed consent was obtained from all individual participants included in the study.

### Design

Eligible patients were invited by their treating oncologist. Informed consent was obtained, after which participants commenced a multidisciplinary intervention consisting of three components: sports medical examinations (SMEs) at the start and end of the intervention, consultations with an oncology occupational physician, and 12 weeks of exercise training. Exercise training sessions consisting of aerobic and strength training took place twice a week and were guided by a physical therapist. The intervention is described in more detail by Groeneveld et al. (2012) [26].

### Assessments

Questionnaires were assessed before (T0) the intervention, and 6 (T1), 12 (T2), and 18 (T3) months after the start of the intervention. The questionnaires were sent and returned by postal mail.

### Measures

#### Dependent variables

The event of interest of the Cox proportional hazard model is time of RTW, defined as length of time in calendar days from the baseline assessment T0. A distinction is made between partial RTW, defined as the initiation of any resumption of work, and full RTW, defined as working the number of hours specified in the labor contract. Censoring was dated at the last assessment at which the participant reported to have not returned to work.

#### Independent variables

Four variables were hypothesized and studied as predictors: perceived work ability, value of work, job self-efficacy, and fatigue. Perceived work ability was assessed with the first question of the Work Ability Index (range 1–10) [28]. We categorized work ability as “low” (1–5) or “adequate” (6–10). Value of work was assessed with one question “Mark the number that reflects the importance of work for you at this moment” (range 1–10, with 1 indicating “not important at all” and 10 “extremely important”). We categorized value of work as “low” (1–5), or “adequate” (6–10). Job self-efficacy was assessed using the 11-item self-efficacy scale developed by Lagerveld, Blonk, Brenninkmeijer, & Schaufeli (2010) [8]. Job self-efficacy was expressed as a mean score (range 1–6) if at least six items were answered, with higher scores reflecting higher self-efficacy. Fatigue was assessed with the general fatigue subscale of the multidimensional fatigue inventory [29]. Responses on each of the four items ranged from 1 (yes, that is true) to 5 (no, that is not true) and were summed (range 4–20). We categorized fatigue as “low” (4–13), or “considerable” (14–20), based on a median score of 14 on the baseline assessment.

For each hypothesized predictor variable, one time-dependent covariate (COV(T)) was composed of the three different assessments (COV.T0, COV.T1, and COV.T2). Time (T), T1, and T2 were defined as the number of days from the baseline assessment T0. In this time-dependent covariate, an assessment of a covariate applies until the next assessment is filled in, which then replaces the previous assessment. Correspondingly, the following syntax was used in SPSS to compose COV(T), with logical statements bold-faced:

$$ \mathrm{COV}\left(\mathrm{T}\right)={\left(\mathbf{T}\le \mathbf{T1}\right)}^{\ast }\ \mathrm{COV}.\mathrm{T}0+{\left(\mathbf{T}>\mathbf{T1}\&\mathbf{T}\le \mathbf{T2}\right)}^{\ast }\ \mathrm{COV}.\mathrm{T}1+{\left(\mathbf{T}>\mathbf{T2}\right)}^{\ast }\ \mathrm{COV}.\mathrm{T}2 $$

#### Confounding variables

Two types of confounding effects were anticipated: prognostic factors (sociodemographic and work-related variables measured at baseline) and three timing variables. The following prognostic factors were studied: age, educational level (certificates or degrees in secondary vocational education or higher were categorized as “high”), bread winner status (categorized as sole breadwinner versus no or shared breadwinner), physical demands of job (item of the VBBA, categorized as scoring “never” versus “sometimes” to “always” [30]). Timing variables were days between initiation of the study with respect to (1) cancer diagnosis, (2) first day of sick leave, and (3) start of chemotherapy.

#### Analysis plan

The data were analyzed in two steps. First, preliminary analyses were performed to study and account for missing data and confounding effects. Second, Cox proportional hazard analysis [27] was performed to assess the predictive significance of four covariates for partial and full RTW as events of interest. To account for potential change of the hypothesized covariates, these were included as time-dependent covariates. All statistical analyses were performed in SPSS version 24.

#### Missing data

Kaplan-Meier plots were used to evaluate the relation of time to partial or full RTW with missing data at the sports medical examination, at T3, or at two assessments.

Missing dates were imputed manually: dates at which the baseline questionnaire (T0) was filled out (*N* = 10) were imputed manually either by the date the form was received by the researcher, or centered between signing informed consent and the first SME. Missing values for first sick leave (*N* = 8) were imputed as 2 months before informed consent was signed.

Other missing data (days between T0 and T1, days between T1 and T2, breadwinner status, work ability, self-efficacy, value of work, fatigue at T0, T1, and T2,) were imputed 20 times by means of predictive mean matching, which is a multiple imputation method similar to regression methods, but relies less on the parametric assumptions of the imputation models [31]. The imputed datasets were used for hypothesis testing, and not for studying confounding effects. More extensive information on the multiple imputation procedure is given in the Online Resource 2.

#### Accounting for confounding effects

Prognostic factors were checked for confounding significance on time to RTW by means of Kaplan-Meier curves and log-rank tests: age, educational level, status as sole breadwinner, and physically strenuous work. Additionally, timing variables were checked for confounding significance for time to RTW. To limit the amount of factors per event, the most significant prognostic factor and the most significant timing variable were selected by means of the lowest *p* value for partial and full RTW separately.

The hypothesized covariates (perceived work ability, value of work, self-efficacy for RTW, fatigue) assessed at baseline were checked for multicollinearity (variance inflation factor (VIF) < 4) with the included prognostic factors, and for each assessment separately. In case VIF > 4, a sensitivity analysis was performed. Moderate and stronger correlations (*r* > .3) among the covariates were reported.

#### Proportional hazard analyses

**Fig. 1:**
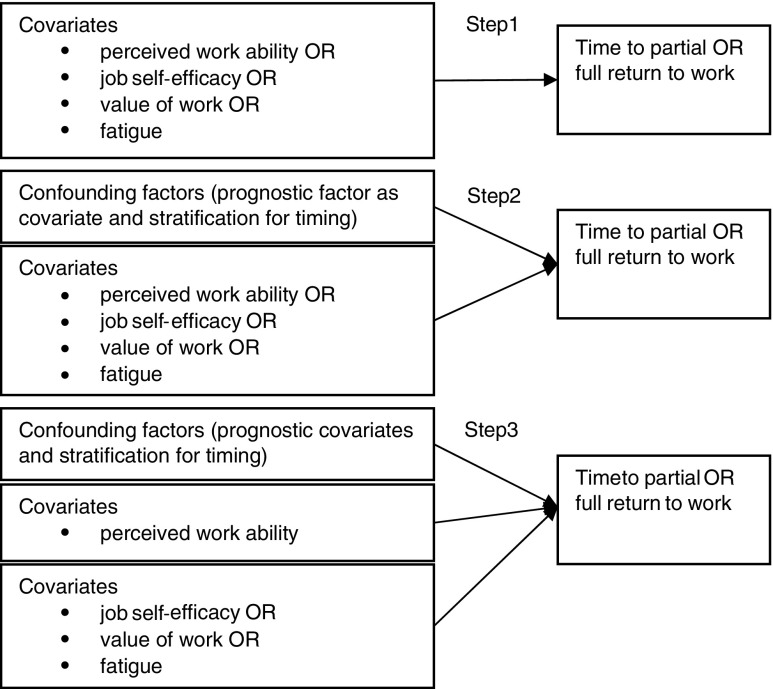
Cox proportional hazard model. Steps 1 and 2 are performed to study research question 1; step 3 is performed to study research question 2

To answer the first research question, two steps were performed. First, univariate, time-dependent Cox proportional hazard models were run for each time-dependent covariate. Second, an adjusted model for each time-dependent covariate was run, to control for potentially confounding effects of timing and prognostic factors. In the third step, the second research question was answered, on the additional predictive value of job self-efficacy, value of work, and fatigue over work ability. Those factors that were significant predictors at *p* values below .1 in the first two steps were added to a model with confounding factors and work ability as covariates. All three steps were performed for both partial and full RTW and are depicted in Fig. 1.

Level of significance was set at .05. Effects of the covariates in both models are presented in terms of hazard ratios with 95% confidence intervals (CI). A hazard ratio > 1 reflects an increased hazard, thus earlier RTW.

## Results

### Participants

Of the 95 participants that were originally included and completed the intervention [32], seven were excluded for the current analyses: two participants never returned the baseline questionnaire, two participants had not reported sick at work, two participants were jobless when they completed the baseline questionnaire, and one participant had fully returned to work before the baseline assessment. Of the remaining 88 participants, seven were censored before the first event as they did not return any of the follow-up assessments, thus were not analyzed. Therefore, for full RTW, 81 cases were analyzed with 56 events of full RTW. Table 1 shows the participant characteristics of all 81 participants that were included in the analyses for full RTW. For partial RTW, of these 81 participants, another five cases were dropped, because they had partially returned to work before the baseline assessment. Therefore, 76 cases were analyzed with 70 events of returning to work partially.

**Table 1:** Participant characteristics

Characteristic	Number (%) or mean (standard deviation)
Sociodemographic factors	
Female	75 (93%)
Age (years)	48.1 (7.3)
Education	
Low	11 (14%)
Intermediate	28 (35%)
High	42 (52%)
Breadwinner status (*N* = 80)	
Sole	38 (48%)
Shared	16 (20%)
Partner	26 (33%)
Disease-related variables	
Cancer type (*n* (%))	
Mamma	70 (87%)
Colorectal	7 (9%)
Non-Hodgkin lymphoma	4 (5%)
Days since diagnosis (M (SD)) (*N* = 73)	80 (42)^a^
Days since first chemotherapy (M (SD)) (*N* = 76)	12 (23)^a^
Chemotherapy started after the baseline assessment (*N* = 76)	23 (30%)
Adjuvant chemotherapy (*n* (%))	71 (88%)
Treatments additional to chemotherapy	
Surgery	72 (89%)
Radiotherapy	25 (31%)
Hormone treatment	10 (12%)
Radiotherapy and hormone treatment	28 (35%)
Work-related variables	
Days since first day of sick leave (*N* = 73)	78 (55)^a^
Type of contract (*N* = 80)	
Permanent employment	67 (84%)
Temporary employment	4 (5%)
Self-employed/other	8 (10%)
Weekly working hours (M (SD)) (*N* = 75)	28.5 (9.9)
Years in current employment (*N* = 80)	10.9 (8.5)
Years in paid employment (*N* = 78)	22.8 (9.6)
Works at large company (> 100) (*N* = 78)	49 (63%)
Shift work/irregular service (*N* = 80)	12 (15%)

*N* = 81 unless stated otherwise

^a^Number of calendar days before the baseline assessment

### Missing data

Percentages of missing data of the covariates are reported in Online Resource 1. None of the missing data patterns were associated with time to RTW.

### Accounting for confounding effects

To control for confounding effects of differences in prognostic factors and timing, the following variables were included as covariates in the models of steps 2 and 3 of the main analyses: sole breadwinner status for time to partial RTW and low educational level for time to full RTW. Additionally, we stratified for time since first chemotherapy in the model of partial RTW and for time since first sick leave in the model of full RTW. Other factors were not statistically significant predictive for RTW. All are reported in the Online Resource 1.

No collinearity was present among the confounding factors and covariates assessed at T0. However, correlations among job self-efficacy, work ability (1–10), and value of work were moderate (0.318 < *r* < 0.462, see Online Resource 1).

### Proportional hazard analyses

All hypothesized time-dependent covariates (perceived work ability, job self-efficacy, value of work, and fatigue) were related with earlier RTW (*p* < .1), and thus in step 3 entered in multivariate models with work ability. Tables 2 and 3 present the hazard ratios for partial and full RTW, respectively. Only job self-efficacy was significant in predicting full RTW (HR (95% CI) = 1.681 (1.048 to 2.696)) in a model that also included perceived work ability.

**Table 2:** Hazard ratios of work-related factors and fatigue on partial return to work

	Univariate (step 1)		Adjusted (step 2)^a^		Multivariate, adjusted (step 3)^a, b^		
	HR	95% CI	HR	95% CI	HR	95% CI	Χ^2^(*p* value)^c^
Confounding factors							
Sole breadwinner (reference: no or shared breadwinner)	1.812	1.013–2.977	–	–	–	–	–
Covariates							
Low work ability (reference: “adequate” work ability)	0.540	0.324–0.900	0.501	0.296–0.848	–	–	–
Higher job self-efficacy (increase in one point)	1.446	0.999–2.094	1.536	1.034–2.282	1.329^d^	0.877–2.014	2.034 (.174)
Low value of work (reference: “adequate” value of work)	0.570	0.345–0.939	0.567	0.338–0.950	0.624	0.369–1.055	3.494 (.076)
Low fatigue (reference: considerable fatigue)	1.753	1.044–2.943	1.631	0.948–2.870	1.443	0.823–2.529	1.824 (.200)

An HR (hazard ratio) > 1 reflects a shorter time to return to work. *CI* confidence interval

^a^Adjustments were stratification for time since first chemo, and including breadwinner status as predictor

^b^Effect over perceived work ability

^c^Improvement of the model by adding the covariate mean of 20 imputations

^d^HR of work ability was not statistically significant in this model

**Table 3:** Hazard ratios of work-related factors and fatigue on FULL return to work

	Univariate (step 1)		Adjusted (step 2)^a^		Multivariate, adjusted (step 3)^a^		
	HR	95% CI	HR	95% CI	HR	95% CI	Χ^2^(*p* value)^b^
Confounding factors							
Low educational level (reference: medium or high)	2.405	1.195–4.838	–	–	–	–	–
Covariates							
Low work ability	0.435	0.244–0.778	0.420	0.229–0.770	–	–	–
Higher job self-efficacy	1.856	1.222–2.817	1.934	1.260–2.968	1.681^c^	1.048–2.696	6.145 (.025)
Low value of work	0.564	0.293–1.084	0.467	0.237–0.918	0.572	0.282–1.160	2.913 (.110)
Low fatigue	2.335	1.279–4.261	2.254	1.230–4.130	1.802	0.937–3.467	3.951 (.074)

*HR* hazard ratio, *CI* confidence interval

^a^Adjustments were stratification for time since first sick leave, and including education as predictor.

^b^Improvement of the model by adding the covariate mean of 20 imputations.

^c^HR of work ability was not statistically significant in this model

## Discussion

This study aimed at identifying potentially modifiable factors that predicted earlier RTW in cancer survivors who received chemotherapy; both univariately, and in addition to perceived work ability. Univariate models showed that adequate perceived work ability, higher job self-efficacy, adequate value of work, and low fatigue predicted earlier partial as well as full RTW. Job self-efficacy predicted earlier full return of work statistically significantly in a multivariate model that also included perceived work ability.

To the best of our knowledge, this study is the first to explicitly acknowledge the variable character of prognostic factors when studying its relation with work resumption in cancer patients. Such an approach corresponds well with our research goal, as we explicitly aimed to focus on potentially modifiable factors. Also, this analysis has the advantage of having more up-to-date estimates of the hypothesized factors as opposed to the standard methods (i.e., survival analysis or Cox regression), thus resulting in more precise estimations of the effects.

Notwithstanding the differences in methods, our results are largely in agreement with previous findings in the literature. Our study showed that work ability was predictive of time to partial RTW. Similar effects were found in other studies. Work ability at 6 months post diagnosis was predictive for earlier work resumption in a sample of 195 persons with mixed cancer diagnoses [6] and was associated with employment status of 50 persons with colorectal cancer [9]. All three studies indicate that work ability is a relevant aspect in occupational rehabilitation for patients with a cancer diagnosis.

Similarly, we found that higher job self-efficacy was related with earlier RTW and was a stronger predictor for full RTW than work ability. In the literature, similar results have been described in a non-cancer population. Job self-efficacy and increases thereof were predictive for earlier full RTW in common mental health disorders [33]. Contrasting, in colorectal cancer patients, job self-efficacy was related with duration of sick leave cross-sectionally at baseline, but not 6 months later. In a multivariate model, job self-efficacy at baseline was also not significantly predictive of employment status 6 months later [9]. Whether patients received chemotherapy was the strongest predictor of employment status in that model, which could explain differences with the current findings. In particular, the current study included only participants who received chemotherapy.

Value of work was related with partial and full RTW in this study, but not in a model that also included work ability. In a prospective cohort study among breast cancer patients [10], importance of work was associated with work ability. That result is congruent with the current findings and strengthens value of work as a relevant aspect for occupational rehabilitation.

Our findings on the influence of fatigue on RTW correspond well with the literature, although interpretation remains complex. Fatigue was a predictor for both partial and full RTW, but was no significant predictor of earlier RTW in a model that also included work ability. Similar to the current results, fatigue was not predictive for earlier partial RTW in a multivariate model that also included work ability [6]. These results could suggest that fatigue may be secondary in predicting time to RTW compared to work ability, with potentially varying impact in different populations. In particular, work ability could mediate the effect that fatigue has on return to work, which would explain why any “direct effect” of fatigue is not present when also work ability is included as predictor.

Please note that the confounding effect of educational level in our study contrasts earlier studies, in which higher education was related with earlier RTW [3, 18, 34] or was no predictor [35]. As educational level was not a focus of this study, the concerning findings should be interpreted with care. A cautious explanation could be an increased pressure to RTW for those with a lower education. In the Netherlands, the ratio of flexible contracts generally increased in the last decade, with increased odds for a flexible contract for those with a lower education [36].

### Implications for practice

The current findings provide multiple leads for enhancing participation of and improving care for cancer survivors. As job self-efficacy was found to be a primary predictor for time to full RTW, monitoring the patient’s job self-efficacy and its development seems relevant for the early detection of barriers for work resumption. The items of the job self-efficacy list provide specific points for vocational guidance of the occupational physician or referral to other interventions.

As value of work was predictive of earlier work resumption, also interventions that increase value of work are deemed effective. In a previous study, value of work was positively related with social support from supervisor and colleagues [10]. As such, social support in the workplace is a relevant subject for intervention to enhance RTW from the employer’s perspective [37]. From the patient’s perspective, empowering cancer patients in communication and negotiation can actually help them in their process of returning to work [11]. Such skills will not only support patients practically in managing RTW [37], but will benefit also through increased importance of work. Additionally, in occupational care for cancer survivors, assuming the desirability of RTW as it was pre-diagnosis should be avoided, and the individual’s meaning and significance of work should be acknowledged [11].

### Strengths and limitations

We would like to emphasize three strengths of this paper. As mentioned earlier, firstly, all studied predictors for RTW are modifiable, thus relevant for designing interventions. A second strength of this paper is the time-dependent character of the covariates, allowing the use of relatively recent estimates of the covariates for estimating proportional hazard. As such we could refrain from performing multiple tests for each assessment in time. We assume that, with a grid of 6 months, still some variation of the covariates in time was ignored. Nevertheless, we consider the analysis of this model a step in the right direction. Thirdly, results can be generalized in view of a fairly homogeneous sample: most participants (87%) were diagnosed with breast cancer and all endured chemotherapy. Chemotherapy is a treatment modality that is associated with reduced work ability [38], has showed to be predictive for later RTW [6, 25], and has been referred to as an important predictor for not working and having a low work ability in colorectal cancer [9].

Some limitations should be kept in mind for the interpretation of the current findings. First, all participants knowingly assigned to an intervention study that included exercise training and consultations with an occupational physician. Such a specific selection could limit the generalizability of the current results, as perceived relevance of the studied constructs is likely high. Second, as in most observational studies, this study suffers from many sources of noise, such as type of contract, physical demands of the job, or duration and modalities of cancer treatment, but has very little options to adjust for it. One particular source of heterogeneity is the large variation in the time since diagnosis (SD = 42 days) and first chemotherapy (SD = 23 days). This implies that there were meaningful differences between participants’ stages in the trajectories through diagnosis, treatment, and rehabilitation. Although we aimed to adjust for the most prominent confounding factors in the models of partial and full RTW separately, it was not feasible to adjust for all potentially confounding factors. Third, it was not specified what the volume of partial work resumption had been, nor could be derived whether the events of full work resumption represented the start of sustainable work resumption. Consequently, the results should be interpreted as modest indications rather than convincing evidence.

### Future directions for research

Perceived work ability and job self-efficacy seem to be key predictors for RTW; therefore, it should be tested what (potentially modifiable) factors contribute to both concepts. A first lead from the current results would be to study if work ability and/or job self-efficacy mediate the relation of value of work and fatigue with earlier RTW.

## Conclusions

Lower fatigue, higher value of work, job self-efficacy, and perceived work ability predicted earlier RTW. Job self-efficacy, but not fatigue or value of work, was of additional predictive value for time to full RTW in addition to perceived work ability. These results provide multiple leads for enhancing work resumption of cancer survivors.

## Electronic supplementary material

ESM 1 (PDF 179 kb)

ESM 2 (PDF 113 kb)

ESM 3 (PDF 139 kb)
