# Supplementary material for: Predictors for earlier return to work of cancer patients
Source: J Cancer Surviv. 2017 Oct 26;12(2):169–77. doi: 10.1007/s11764-017-0655-7 (PMC5884890; doi:10.1007/s11764-017-0655-7)
Supplement: Supplementary file 1 — (PDF 179 kb) [file 11764_2017_655_MOESM1_ESM.pdf]

Online Resource 1

Article title: Predictors for earlier return to work of cancer patients

Authors: Wolvers MDJ, Leensen MCJ, Groeneveld IF, Frings-Dresen MHW, De Boer AGEM

Journal: Journal of Cancer Survivorship

Corresponding author: A.G.E.M. de Boer, Academic Medical Center, department: Coronel Institute of Occupational Health, Amsterdam Public Health research institute, Amsterdam, the Netherlands (a.g.deboer@amc.uva.nl | +31 20 566 5323)

Additional results on preliminary analyses

Missing data

The amount of missing data of the independent variables on the three assessments (baseline, six months (T1), 12 months (T2)) is presented in Table 4.

Table 1. Missing information of covariates

	fraction missing data at baseline	fraction missing data at T1	fraction missing data at T2
Work ability	0.03	0.13	0.24
Job self-efficacy	0.07	0.16	0.25
Value of work	0.01	0.13	0.24
Fatigue	0.06	0.15	0.24

Potential confounders

Both prognostic factors and timing variables were studied separately for confounding effects on partial and full return to work. To limit the number of 'events per factor', the most significant predictor (as indicated by the lowest *p*-value) in each confounder category was used to adjust the analyses: one prognostic factor was included as covariate and one timing variable was used for stratification. All results are presented in Table 5.

Figure 3 shows the stratified survival plots of the selected confounding factors.

Table 2. Predictive value of prognostic factors and timing

	**Partial RTW**		**Full RTW**	
	HR (/log-rank test)	*p*-value	*HR*	*p*-value
**Prognostic factors**				
age (years)	1.035	.067[b]	1.034	.109
- age < 45[a]	0.659	.166	0.831	.571
Education (3 cat)	(CHI^2 =1.401)	.496	(CHI^2 =7.247)	.027
- education low (2 cat)	1.167	.653	*2.316*	*.018*
NO physically demanding job	1.122	.634	0.732	.248
NOT 'sole breadwinner'	*0.523*	*.008*	0.979	.938
**Timing, days since**				
diagnosis (days)	1.003	.436	0.997	.327

- days < 76 (median)	(CHI^2 = 0.471)	.493	(CHI^2 = 0.350)	.554
- days < 100	(CHI^2 = 0.076)	.782	(CHI^2 = 2.045)	.153
- days < 100	.925	.783	1.618	.157
first day of sick leave (days)	1.004	.075	0.997	.222
- days < 90	(CHI^2 = 0.833)	.361	*(CHI^2 = 3.650)*	*.056* [b]
- days < 90	0.793	.363	1.793	.060 [b]
first chemotherapy (days)	*1.011*	*.038*	1.006	.299
- days < 0	(CHI^2 = 1.970)	.160	(CHI^2 = 0.117)	.733
- days < 14	(CHI^2 = 2.742)	.098	(CHI^2 = 1.268)	.260
- days < 0	1.474	.152	1.110	.733

Note: RTW is return to work. The 'most significant' prediction of all potential confounders in each cell is in *italics*. These analyses were performed on the original dataset, so cases were excluded in case of missing data. [a] Split in accordance with Roelens et al. [b] Does not satisfy proportional hazard assumption.

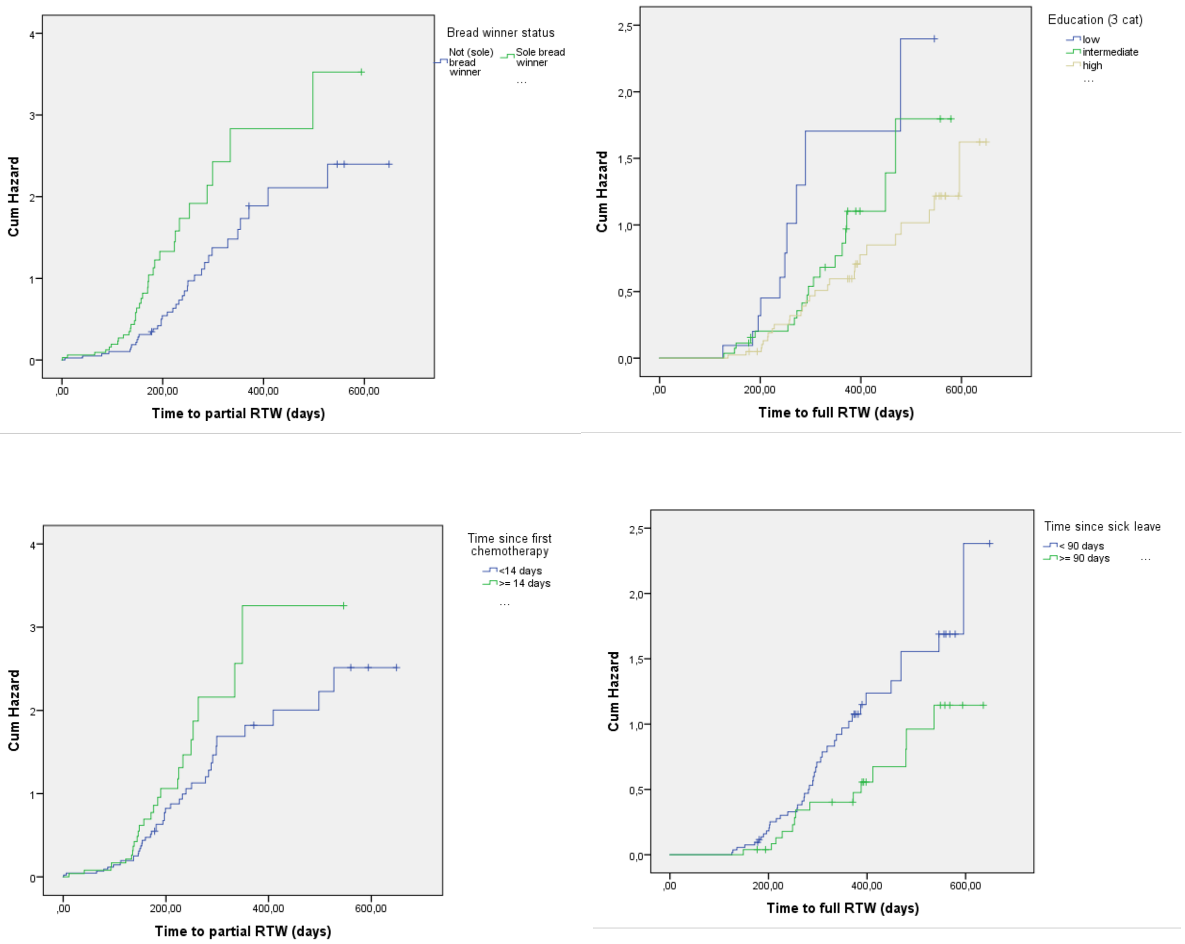

Figure 1. Hazard functions of confounding factors. Partial return to work (RTW) was adjusted for breadwinner status and time since first chemotherapy. Full RTW was adjusted for educational level and time since first sick leave. Stratified cumulative hazard plots for education represent three categories, however a dummy representing low vs. intermediate AND high education was used to adjust analyses of full RTW.
