# Supplementary material for: Predictors for earlier return to work of cancer patients
Source: J Cancer Surviv. 2017 Oct 26;12(2):169–77. doi: 10.1007/s11764-017-0655-7 (PMC5884890; doi:10.1007/s11764-017-0655-7)
Supplement: Supplementary file 2 — (PDF 113 kb) [file 11764_2017_655_MOESM2_ESM.pdf]

Online Resource 2

Article title: Predictors for earlier return to work of cancer patients

Authors: Wolvers MDJ, Leensen MCJ, Groeneveld IF, Frings-Dresen MHW, De Boer AGEM

Journal: Journal of Cancer Survivorship

Corresponding author: A.G.E.M. de Boer, Academic Medical Center, department: Coronel Institute of Occupational Health, Amsterdam Public Health research institute, Amsterdam, the Netherlands (a.g.deboer@amc.uva.nl | +31 20 566 5323)

Multiple imputations

To prevent that cases are discarded due to missing data, we produced 20 datasets in which missing data points were imputed with different values, using fully conditional specification[1] (also known as chained equations or MICE) in SPSS, version 24. Multinomial logistic regression was used for categorical variables and the predictive mean matching (PMM) method for scale variables, following recommendations from Marshall et al. (2010) [2][39] . PMM is similar to regression methods, but relies less on the parametric assumptions of the imputation models as the observed value that is closest to the value drawn by the imputation model is imputed.

After convergence of the models was visually inspected using the trace plots of two imputations with 500 iterations each, the number of iterations was set at the SPSS default of 10. Of some variables, up to 19% of the information was missing (Table 6), therefore 20 imputed datasets were generated, following recommendations to prevent loss of power[3] and to have optimal reproducibility. [4]

Table 1. Multiple imputation prognostics

	fraction missing information in univariate time-dependent model PRTW	fraction missing information in univariate time-dependent model FRTW
Work ability	.046	.102
Fatigue	.048	.185
Self-efficacy	.116	.191
Value of work	.079	.123

Imputed variables

Missing data (work ability, self-efficacy, value of work, fatigue at T0, T1, and T2, number of days between T0 and T1, days between T1 and T2, and breadwinner status) were imputed. Data were imputed as raw scores (work ability, value of work) or sum scores (self-efficacy, fatigue).

[1] Van Buuren 2007 Stat Methods Med Res **16**: 219–242.
[2] Marshall, Altman, and Holder 2010. BMC Med Res Methodology **10**:112.
[3] Graham, Olchowski, and Gilreath 2007. Prev Sc **8**: 206-13.
[4] White, Royston, and Wood 2011. Statistics in Med **30**: 377-99.

Auxiliary variables

'MICE procedures assume that the data are Missing At Random. While it is almost always impossible to test this assumption, including auxiliary variables (in the imputation regression model) that are predictive of missingness as well as variables that are correlated with variables that will be used in the data analysis stage, can reduce bias and make the MAR assumption more plausible.'[5]

All imputed variables also served as predictor variables in the imputation models for other imputed variables. Additional parameters that were included in all imputation models were perceived work ability, job self-efficacy, value of work, and fatigue at T3, age, maximal oxygen consumption at T0 (assessed on a bicycle ergometer by a sports physician), time since diagnosis, time since first sick leave, time since first chemotherapy, and the sum scores of the fatigue subscale of the EORTC-QLQ-30 at T0 to T3.[6]

Pooling

Analyses were performed on each dataset and the results were pooled into one parameter according to 'Rubin's Rules'. Only pooled estimates are reported.

[5] Azur et al. 2012. Int J Methods Psychiatr Res **20**: 40-9.
[6] Aaronson et al. 1993. J Nat Canc Inst **85**: 365-76.
