# Supplementary material for: Predictors for earlier return to work of cancer patients
Source: J Cancer Surviv. 2017 Oct 26;12(2):169–77. doi: 10.1007/s11764-017-0655-7 (PMC5884890; doi:10.1007/s11764-017-0655-7)
Supplement: Supplementary file 3 — (PDF 139 kb) [file 11764_2017_655_MOESM3_ESM.pdf]

Online Resource 3

Article title: Predictors for earlier return to work of cancer patients

Authors: Wolvers MDJ, Leensen MCJ, Groeneveld IF, Frings-Dresen MHW, De Boer AGEM

Journal: Journal of Cancer Survivorship

Corresponding author: A.G.E.M. de Boer, Academic Medical Center, department: Coronel Institute of Occupational Health, Amsterdam Public Health research institute, Amsterdam, the Netherlands (a.g.deboer@amc.uva.nl | +31 20 566 5323)

Sensitivity analyses

Sensitivity analysis of multiple imputations

Correlates among the hypothesized covariates assessed at baseline, T1, and T2 were performed for both the original data (complete case only) and imputed datasets. At baseline, not a lot data was missing, and differences among estimates are small. At T2, correlation estimates from the pooled dataset deviate slightly towards zero. The largest difference is seen for self-efficacy (r = .731 compared to .543).

Table 1. Correlation matrix of imputed variables at T0 (Spearman's r, *p*-value)

	work ability		value of work		SE RTW	
	pooled	complete cases	pooled	complete cases	pooled	complete cases
work ability	1	1	-	-	-	-
value of work	0.323 (.002)	0.318 (.003)	1	1	-	-
self-efficacy	0.425 (<.001)	0.462 (<.001)	0.336 (.002)	0.352 (.001)	1	1
fatigue	-0.189 (.083)	-0.131 (.233)	-0.222 (.042)	-0.179 (.096)	-.205 (.061)	-.209 (.058)

Note: The dichotomized scores are used here when applicable. N of complete cases ranges between 82 and 88, N of the pooled imputed datasets (20 imputations) is 89.

Table 2. Correlation matrix of imputed variables at T1 (Spearman's r, *p*-value)

	work ability		value of work		SE RTW	
	pooled	complete cases	pooled	complete cases	pooled	complete cases
work ability	1	1				
value of work	.395 (<.001)	.462 (<.001)	1	1		
self-efficacy	.559 (<.001)	.646 (<.001)	.423 (<.001)	.483 (<.001)	1	1
fatigue	-.453 (<.001)	-.503 (<.001)	-.290 (.010)	-.337 (<.001)	-.483 (<.001)	-.537 (<.001)

Note: The dichotomized scores are used here. N of complete cases ranges between 74 and 77, N of the pooled imputed datasets (20 imputations) is 89.

Table 3. Correlation matrix of imputed variables at T2 (Spearman's r, *p*-value)

	work ability		value of work		SE RTW	
	pooled	complete cases	pooled	complete cases	pooled	complete cases
work ability	1	1				
value of work	.143 (.244)	.205 (.093)	1	1		
self-efficacy	.373 (.006)	.504 (<.000)	.295 (.030)	.388 (.001)	1	1
fatigue	-.298 (.012)	-.312 (.010)	-.189 (.147)	-.258 (.035)	-.315 (.013)	-.404 (.001)

Note: Dichotomized scores are used here when applicable (so for work ability, value of work, and fatigue). N of complete cases ranges between 66 and 68, N of the pooled imputed datasets (20 imputations) is 89.

Sensitivity analysis of dichotomization

To show the difference between correlations among raw and dichotomized variables, Table 10 was added. Comparison of Table 10 with Table 9 shows that correlations of raw scores are generally slightly higher compared to correlations among dichotomized variables.

Table 4. Correlation matrix of raw imputed variables at T2 (Spearman's r, *p*-value)

	work ability	value of work	SE RTW
	complete cases	complete cases	complete cases
work ability	1		
value of work	0.365 (.002)	1	
self-efficacy	0.731 (.000)	0.459 (.000)	1
fatigue	-0.629 (.000)	-0.282 (.021)	-0.471 (.000)

Note: The sum scores/raw scores and not the dichotomized scores are used here. N ranges between 66 and 68.
